# Anxiety and depressive symptoms in patients with psoriasis

**DOI:** 10.1192/j.eurpsy.2021.485

**Published:** 2021-08-13

**Authors:** R. Sallemi, M. Bouhamed, R. Masmoudi, I. Feki, J. Masmoudi

**Affiliations:** Psychiatry, Hedi chaker hospital, Sfax, Tunisia

**Keywords:** Anxiety, depressive, psoriasis

## Abstract

**Introduction:**

Psoriasis is a chronic inflammatory skin disease that affects approximately 2% of the population. It seems to have a multifactorial aetiology and it can be considered as a psychosomatic disorder.

**Objectives:**

To determine risk factors for anxiety and depression in psoriasis

**Methods:**

Case-control study including 44 subjects with psoriasis and 50 controls without psoriasis. All participants answered the Hospital Anxiety and Depression Scale (HADS) to measure the severity of anxiety and depression

**Results:**

Descriptive study: We solicited 44 patients and the average age was 45.8 years. The majority of patients were married (70.5%), unemployed (40.5%), without medical heredity (84,6%). Psoriasis was in plaque (65.9%), guttate (20.5%), pustular (13.6.5%).Its severity, assessed by BSA, was mild to moderate in 72.7% of cases and associated arthropathy was noted in 29.5% of patients.the prevalences of anxiety and depression estimated at 29.54% and 18.18% respectively. Analytical study: Subjects with psoriasis, as opposed to controls, showed higher levels of anxiety (29,54% vs 15,9%) and depression (18,18% vs 4,54%) but there was no significant difference (p=0,335, p=0,573) Depression was significantly more important for single (p=0.043), for patients with associated arthropathy (=0.005) and for guttate form (p=0.015) According to the severity of the disease: patients with mild disease are more anxious and patients with severe disease are more depressed

**Conclusions:**

Higher scores in anxiety and depression is common in psoriasis. Dermatologists should give special attention to this subgroup of persons with psoriasis in order to prevent future psychopathology.

